# Towards Omni-Tomography—Grand Fusion of Multiple Modalities for Simultaneous Interior Tomography

**DOI:** 10.1371/journal.pone.0039700

**Published:** 2012-06-29

**Authors:** Ge Wang, Jie Zhang, Hao Gao, Victor Weir, Hengyong Yu, Wenxiang Cong, Xiaochen Xu, Haiou Shen, James Bennett, Mark Furth, Yue Wang, Michael Vannier

**Affiliations:** 1 Biomedical Imaging Division, VT-WFU School of Biomedical Engineering and Sciences, Blacksburg, Virginia Tech, Blacksburg, Virginia, United States of America; 2 Department of Radiology, University of Kentucky, Lexington, Kentucky, United States of America; 3 Department of Mathematics, University of California Los Angeles, Los Angeles, California, United States of America; 4 Department of Medical Physics and Radiation Safety, Baylor Health Care System, Dallas, Texas, United States of America; 5 Biomedical Imaging Division, VT-WFU School of Biomedical Engineering and Sciences, Wake Forest University Health Sciences, Winston-Salem, North Carolina, United States of America; 6 Medical Business Unit, Texas Instruments Inc., Dallas, Texas, United States of America; 7 Comprehensive Cancer Center, Wake Forest University Health Sciences, Winston-Salem, North Carolina, United States of America; 8 Department of Electrical and Computer Engineering, Virginia Tech, Blacksburg, Virginia, United States of America; 9 Department of Radiology, University of Chicago, Chicago, Illinois, United States of America; NIH, United States of America

## Abstract

We recently elevated interior tomography from its origin in computed tomography (CT) to a general tomographic principle, and proved its validity for other tomographic modalities including SPECT, MRI, and others. Here we propose “omni-tomography”, a novel concept for the grand fusion of multiple tomographic modalities for simultaneous data acquisition in a region of interest (ROI). Omni-tomography can be instrumental when physiological processes under investigation are multi-dimensional, multi-scale, multi-temporal and multi-parametric. Both preclinical and clinical studies now depend on *in vivo* tomography, often requiring separate evaluations by different imaging modalities. Over the past decade, two approaches have been used for multimodality fusion: Software based image registration and hybrid scanners such as PET-CT, PET-MRI, and SPECT-CT among others. While there are intrinsic limitations with both approaches, the main obstacle to the seamless fusion of multiple imaging modalities has been the bulkiness of each individual imager and the conflict of their physical (especially spatial) requirements. To address this challenge, omni-tomography is now unveiled as an emerging direction for biomedical imaging and systems biomedicine.

## Introduction

The physiome concept was first presented to the International Union of Physiological Sciences (IUPS) in 1993, and later designated as a strategic area by IUPS in 2001 [1]–[3]. Physiome describes physiological units and their interactions from the genome scale to complex organisms in a systematic fashion. The IUPS Physiome Project supports a worldwide repository of models and datasets, and represents an integral component of systems biomedicine. Biomedical imaging has been instrumental for the Physiome Project, and is yet to be improved for much richer functional, cellular and molecular information.

In the medical imaging field, efforts are being made to link molecular assays with diagnostic imaging [4], [5]; however, success to date has been rather limited. One reason is that current medical imaging scanners do not individually offer a wide enough spectrum of information. For example, current x-ray CT produces a limited amount of information from gray-scale images based on differences in linear attenuation coefficients of various tissues. On the other hand, an information explosion is seen from genetic and epigenetic profiling. This imbalance between phenotypic information (e.g., CT images) and genome-level information (e.g., RNA data) demands more capabilities from the *in vivo* imaging side. Indeed, the medical imaging field is rapidly trending in this direction. Turning again to x-ray CT as an example, the transition has started from gray-scale to true-color images with energy-sensitive, photon-counting detector technology [6]. Another area of advancement is x-ray phase-contrast and dark-field imaging [7], [8]. Overall, both imaging modalities and contrast agents are being rapidly improved.

The holy grail of biomedical imaging is an integrated system capable of producing tomographic, simultaneous, dynamic observations of highly complex biological phenomena *in vivo*. The multimodality fusion, or multimodal fusion, approach has made significant strides towards meeting this challenge, as demonstrated by the popularity of PET-CT and other hybrid systems [9]–[13]. We envision that tomography will evolve beyond current modality fusion and towards grand fusion, a large-scale fusion of many imaging modalities, which is called omni-tomography/multi-tomography [14]. Unlike modality fusion, omni-tomography is for truly simultaneous and often local reconstruction of multiple imaging mechanisms such as CT, MRI, PET, SPECT, US, and optical imaging.

**Figure 1:**
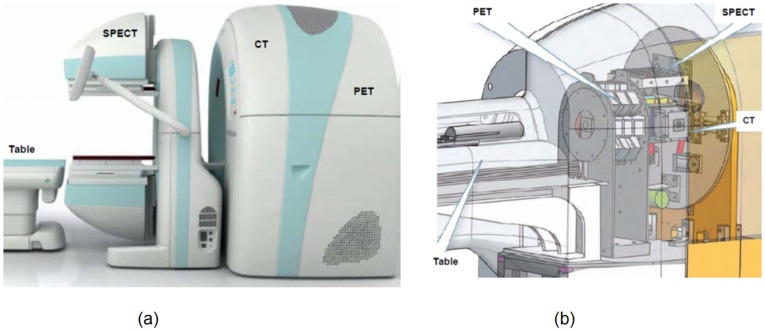
State-of-the-art tri-modality fusion systems. (a) The AnyScan system for clinical PET-SPECT-CT, and (b) the Albira system for preclinical PET-SPECT-CT ((a) and (b) from http://www.mediso.de/anyscan-sc.html and http://www.cmi-marketing.com/7modalities respectively, with the legends added by the authors of this article).

Since the advent of diagnostic imaging, there has been a powerful push to combine imaging modalities in a single coordinate system. Modality fusion began with PET-CT which revolutionized medical diagnosis. A PET-CT scanner sequentially acquires CT and PET images in an integrated gantry. As a result, metabolic processes from PET can be co-registered with anatomic information from CT. As such, PET-CT has redefined the fields of oncology, surgical planning, and radiation therapy. There are several examples of contemporary modality fusion devices. Mediso developed the first human PET, SPECT and CT system AnyScan (http://www.mediso.de/anyscan-sc.html). As shown in Figure 1(a), it provides sequential anatomical and functional imaging within a single framework. Another company, Carestream, has put seven preclinical imaging modalities in two instruments (http://www.cmi-marketing.com/7modalities), covering PET, SPECT, CT, fluorescence, luminescence, radioisotopic and radiographic imaging. As shown in Figure 1(b), the Carestream Albira system is designed for sequential micro-PET and micro-SPECT-micro-CT data acquisitions, quite similar to the Mediso AnyScan system. PET-MRI is the most recent result in the modality fusion field [15]–[17]. For example, PET-MRI is capable of assessing myocardial viability (PET), functions and metabolism (MRI) for diagnosis of cardiac pathology in a single examination.

**Figure 2:**
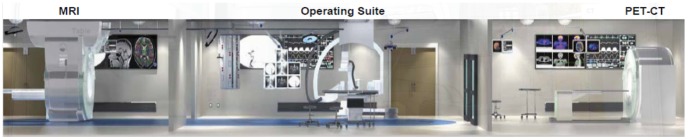
Advanced Multimodality Image Guided Operating (AMIGO) Suite unveiled on May 4, 2011. It is an integrated surgical and interventional environment as the translational test bed of the National Center for Image-Guided Therapy (NCIGT) at the Brigham and Women’s Hospital (BWH) and Harvard Medical School (from http://www.ncigt.org/pages/AMIGO, with the legends added by the authors of this paper).

To go beyond the state of the art in modality fusion, it can be imagined that additional tomographic modalities could be added for simultaneous characterization of biomedical properties [18]–[20]. However, this mission immediately appears impractical due to the conflict of spatial and other physical requirements imposed by scanners. We may longitudinally assemble all involved scanners, but this arrangement would make synchronized capture impossible, especially when relatively fast processes (*e.g*., many physiological phenomena) and relatively slow modalities (*e.g*., PET and SPECT) are involved. The arguments for, and limitations of the classic modality fusion approach are demonstrated in the latest development of the Advanced Multimodality Image Guided Operating (AMIGO) Suite project, shown in Figure 2.

Omni-tomography was inspired and enabled by a recent theoretical breakthrough – interior tomography [21]–[28], which is an approach initially developed for CT but now promoted as a general imaging principle. While classic CT theory targets theoretically exact reconstruction of a whole cross-section or volume from untruncated projections, real-world applications focus often on a region of interest (ROI). A long-standing barrier has been that traditional CT methods cannot exactly reconstruct an ROI solely from truncated projections along x-rays through the ROI. This is the well-known “*interior problem*” which does not have a unique solution in an unconstrained setting. The interior problem and approximate inversion algorithms were extensively studied in the 1980s and 1990s, and the fact that precise image reconstruction cannot be obtained from purely local data contributed to the long-standing architectures of CT and micro-CT scanners whereby detectors must be wide enough to cover a transaxial slice of a patient or animal. This problem also exists for most other tomographic modalities, which were all developed in the same spirit of CT. Over the past several years, the interior problem has been revisited for theoretically exact image reconstruction over an ROI under rather mild practical conditions such as a known sub-region or a piecewise constant or polynomial ROI model [21]–[28]. This advancement means that the data acquisition system can be made rather narrow. More excitingly, this interior approach has been extended for SPECT, MRI, and so on.

To overcome the aforementioned physical conflict for grand fusion, here we propose to transform each relevant imaging modality into a slim or compact imaging component for ROI-targeted data acquisition and image reconstruction. This is in contrast to the traditional untruncated acquisition and global reconstruction. These compressed imaging components can then be integrated into a single gantry for concurrent data acquisition and composite interior reconstruction in a unified framework. In this article, top-level omni-tomographic system architectures are first presented. The technical basis for omni-tomography is then illustrated with interior tomographic reconstructions of representative modalities. Finally, major applications of omni-tomography are discussed, along with promising research directions.

## Results

By our recently proposed interior tomography principle, we have a large flexibility to integrate various imaging modalities when they only target a relatively small ROI. We have systematically analyzed a number of architectures for omni-tomography, and realized that each has advantages and disadvantages. Presently, we focus on a ring-shaped design and a double-magnetic-donut-based design, as two initial examples. In the following, we will emphasize the top-level features instead of the technical details.

### Ring-shaped Design

**Figure 3:**
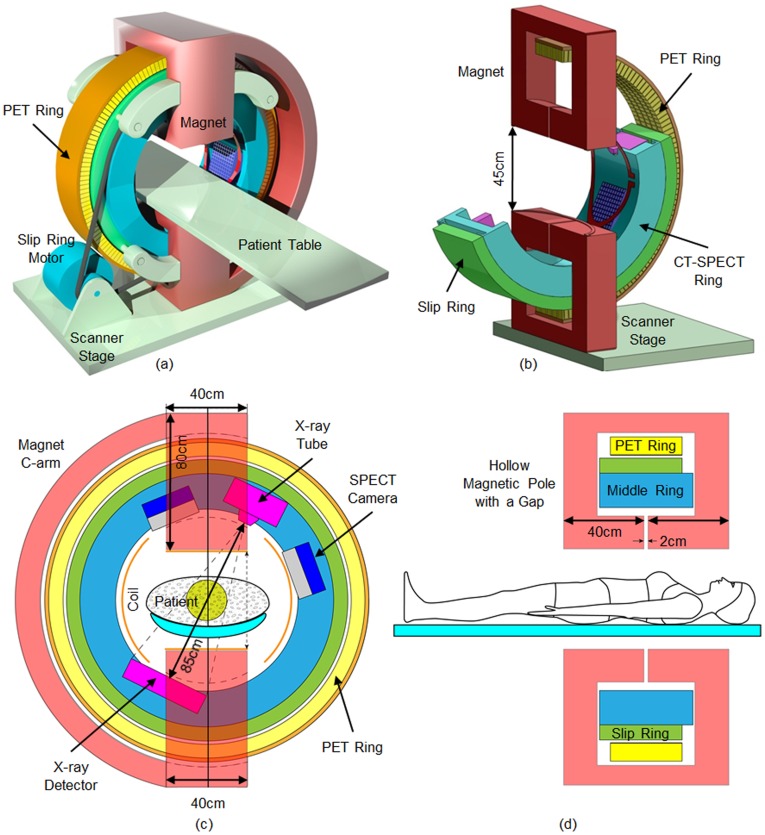
Ring-shaped design for omni-tomography. (a) A 3D rendering of the top-level design, (b) a partial rendering, (c) an in-plane view, and (d) a through-plane view. There are two static rings and one rotating ring for omni-tomography. While the red C-arm is a permanent magnet and the yellow outer ring contains PET crystals, the blue ring supports a CT tube, a CT detector and a pair of SPECT camera. The blue CT-SPECT ring is on a green slip ring (like a large ball bearing) as the interface for power and data. The CT-SPECT ring, the slip-ring, and the PET ring all go through the magnetic poles.

Figure 3 illustrates the ring-shaped system architecture for omni-tomography (additionally Video S1). All the major tomographic modalities are incorporated into three rings: a C-arm-like magnet; a middle ring containing an x-ray tube and a detector array, and a pair of SPECT detectors; and an outer ring for PET. The yoke for N and S poles of the magnet are configured to form a C-arm. The middle ring is designed to enable data acquisition for both interior CT and interior SPECT. This rotating ring is embedded in a slip-ring (similar to a large ball bearing) for power and signal transmission. The rotating ring, the slip-ring, and the PET ring all go through the magnetic poles. The system will easily accommodate a patient with a chest size of 22×35 cm^2^. An ultrasound (US) transducer may be incorporated. In a typical setup, appropriate shielding should be implemented. The modality-specific key features are described as follows.

The MRI component is feasible, as already demonstrated by the commercial open MRI scanners. As shown in Figure 3, the MRI component consists of two permanent magnetic poles. The vertical gap between these poles is 45 cm, and was chosen in a numerical simulation to provide a sufficiently homogeneous local magnetic field of 15–20 cm in diameter in the center of the gantry. The simulation also determined the width (40 cm) and length (2×40 cm) of the magnetic heads. This configuration leaves sufficient space for other modalities. A deviation from the commercial open MRI design is that each magnet pole has a gap of 2 cm to let the middle ring modalities look through the magnet. Hence, the CT tube and detector, as well as the SPECT cameras, can perform full-scans to the extent defined by the gap through the magnet, and cone-beam scans when the magnet is not in the radiation paths.

**Figure 4:**
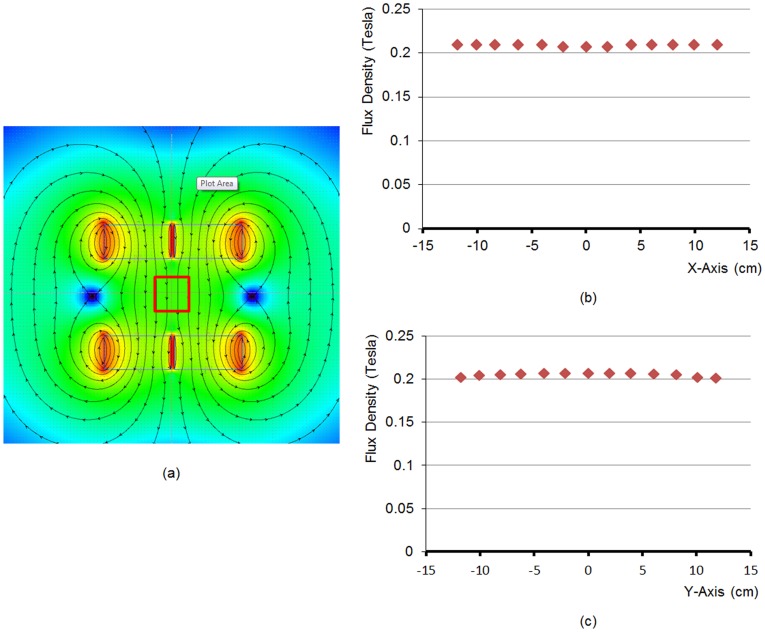
Generation of a locally homogeneous magnetic field. (a) The magnetic flux from four permanent blocks. The square area (20×20 cm^2^) represents a region of interest (ROI) where the magnetic flux ranges from 0.208 to 0.211Tesla; (b) and (c) the magnetic flux plots along the x- and y-axes respectively. Each magnetic block is of 40×40×20 cm^3^, with a gap of 2 cm between two parts of each magnetic pole.

We used the Vizimag software (http://www.vizimag.com) to simulate a locally uniform magnetic field between the poles. The targeted local magnetic field strength was set to 0.2 Tesla. The magnetic field may be adjusted by changing ferromagnetic materials and the dimensions of the magnetic blocks or using an alternative technology. The generated magnetic field is shown in Figure 4. The magnetic flux varies from 0.208 to 0.211 Tesla over an ROI of 20×20 cm^2^ with its origin at the iso-center of the main imaging plane of the omni-tomographic scanner. The field uniformity may be improved with technical refinements. The gradient coils of current open MRI scanners [29], [30] may be modified for omni-tomography. Figure 3 shows a possible configuration of gradient coils. MRI shielding is required, including RF interference shielding, electromagnetic (EM) interference shielding, electromagnetic pulse shielding, and so on. Highly conductive, non-woven electromagnetic shielding materials may be used. These techniques are maturing, and should in principle pose little difficulty [31].

The middle ring of the omni-tomographic system contains x-ray CT and SPECT components. The CT component has a source and a detector array. A typical source configuration includes an x-ray tube (e.g., Varian GS-3074, 23.5×410×13.5 cm^3^), a heat exchanger (e.g., Varian HE 300, 23.5×410×13.5 cm^3^), and a generator (e.g., Spellman 16010, 20.5×40×50 cm^3^). A flat panel (e.g., Varian PaxScan4030CB, 47×37×7 cm^3^) or photon-counting spectroscopic detector array may be used. The source-to-detector distance is approximately 85 cm, consistent with the conventional CT geometry. Interior tomography was originally developed for CT, and has produced excellent results [21], [22], [32], [33]; see the following section for more details. Note that the interior CT scan may be potentially used to estimate the attenuation background over the whole field of view, and enable anatomically-specific attenuation correction for SPECT and PET. In the future, x-ray detectors outside the primary CT beam may be utilized for scattering characteristics.

Two solid-state SPECT cameras are in our design. These cameras are collimated in parallel-beam geometry and arranged orthogonally. The dual-detectors should double the acquisition speed relative to a single detector. There are several commercially available systems (Gamma Medica, GE Triumph) that use CZT detectors. The 16×20 cm^2^ CZT SPECT detector (Gamma Medica, Northridge, CA) is a good candidate for our system. A CZT detector can potentially detect x-ray and gamma-ray photons simultaneously, which is a future possibility. A converging or pinhole collimator may be used when appropriate. Both types magnify features in an ROI. A multi-pinhole collimator is an option when better sensitivity is desired [34], [35]. A diverging collimator can image larger structures with a smaller detector. Furthermore, data compromised when the SPECT cameras are behind the magnetic heads can be fixed by attenuation correction since the magnet is semi-transparent to gamma rays. The rationale for putting the SPECT and CT components on the middle ring, instead of in front of the magnetic heads, is to limit their interference on the magnetic field.

The PET detector ring of 120 cm internal diameter consists of LYSO crystals but it could be built out of CZT or other solid-state materials. In LYSO crystal-based systems, the scintillation emission can be detected with avalanche photodiode detectors (APDs). The detector units are 4×4×20 mm^3^. There are 4 units per detector block, 471 detector blocks per ring, and 20 rings in total. The axial extent is 16 cm. Another part is a coincidence timing or time-of-flight (TOF) analysis circuit, which is commercially available. The 511KeV PET photons are sufficiently energetic to pass through the solid-state CT and SPECT detectors, and reach the PET detector ring. The PET circuitry can be customized for ROI imaging. Prior structural knowledge of the CT and SPECT detectors may be utilized to correct hardware-related attenuation for PET. Positioning the PET ring the furthest away from the MRI system should produce a maximum isolation for the PET detector and electronics from the magnet field. In some existing PET-MRI systems, the PET ring is inside the main magnet ring. For example, the Siemens Biograph PET-MRI system incorporates MRI compatible PET detectors into an MRI system. A number of PET-MRI systems use PET inserts. These designs are fundamentally constrained by the global data acquisition, and cannot reach the same level of hybrid imaging as omni-tomography.

US imaging is cost-effective and widely used. MRI compatible US systems are already commercially available. In our design, US imaging may be easily added, with the transducer and cables shielded [36]. Also, photo-acoustic and thermal-acoustic imaging can be considered in this context.

Optical imaging is important for molecular and cellular information, often aided by fluorescence and bioluminescence probes. Typically, optical imaging has a penetrating depth limitation. Interestingly, x-rays and gamma rays can induce fluorescence and luminescence signals, which can be quite significant when nanoparticles such as gold nanoparticles and nano-phosphors are used in sufficient concentration. There is substantial space in the CT-SPECT ring where fluorescence and/or luminescence cameras may be placed. For example, we can implement interior x-ray fluorescence CT (XFCT) since x-rays are not strongly diffusive [37], [38].

### Double-Magnetic-Donut-Based Design

**Figure 5:**
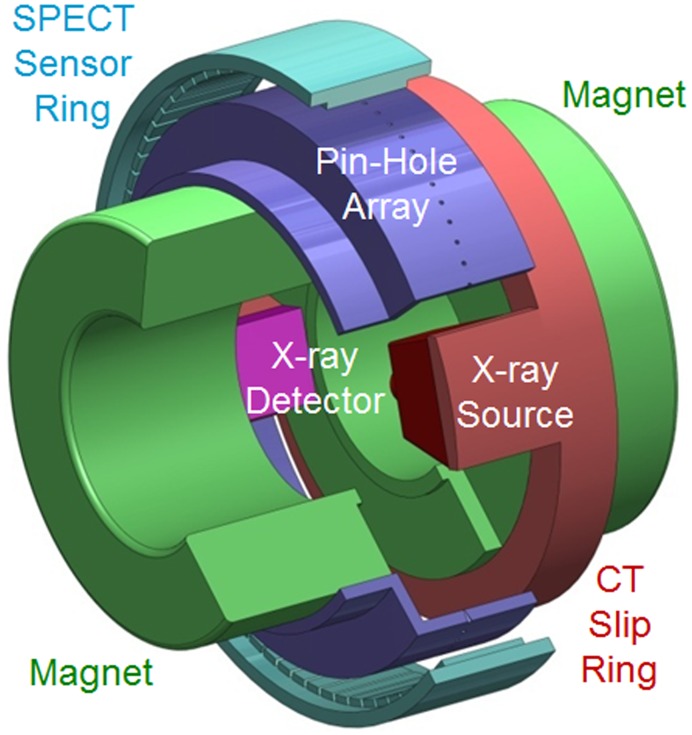
Double-magnetic-donut-based design for omni-tomography. The pair of green magnetic rings is arranged similar to that in the Fonar UPRIGHT Multi-Position MRI (http://www.fonar.com/standup.htm) but with a decreased spatial extent of a homogeneous magnetic background field and thus an increased gantry room for interior CT, interior SPECT, and other modalities. This design is scalable according to preferred sizes of animals or humans.

As another omni-tomographic system design in Figure 5, we can adapt the double-donut-shaped open MRI scanner to integrate (1) an interior CT imager, (2) a stationary multi-view interior SPECT imager in reference to the work by Tsui at Johns Hopkins University, (3) an interior MRI imager based on the design by Fonar, and (4) an optical endoscope for medium-sized animal imaging. With the interior tomography strategy, the data acquisition speed can be much improved, such as with multi-view SPECT. Interior MRI is a major innovation. Two permanent rings will be used to synthesize a homogeneous magnetic field of ∼7 cm in diameter. For physiological studies, we can build a remotely controllable environment (gas exchange, temperature, and various accessories) within the imaging chamber, and measure non-imaging signals.

## Methods

The omni-tomographic architecture is characterized by ROI-oriented data acquisition and quantitative ROI reconstruction using the recently developed interior tomography approach [21]–[28]. In other words, interior tomography is both the theoretical basis and enabling technology for omni-tomography. In this section, we present advanced interior CT, interior SPECT, interior MRI, as well as a unified image reconstruction scheme that uses both CT and MRI data synergistically. The proposed algorithms and simulation results are based on the system architecture shown in Figure 3.

### Interior CT

**Figure 6:**
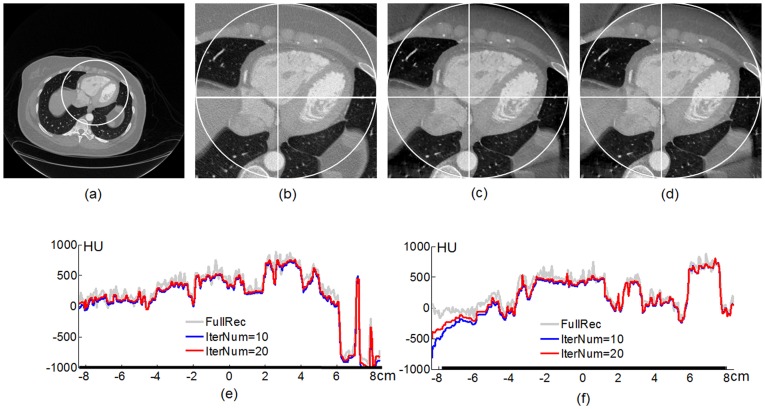
Interior CT reconstruction of a cardiac region from a clinical patient dataset collected on a GE Discovery CT750 HD scanner. (a) The reference image reconstructed from global projections using the conventional filtered backprojection (FBP) method, (b) a magnified interior cardiac region, (c) and (d) the interior reconstructions from truncated local projections after 10 and 20 iterations, respectively. (e) and (f) The profiles along the horizontal and vertical white lines respectively in (b)–(d), where the thick lines on the horizontal axes indicate the ROI. The display window for (a)–(d) is [−1000, 1000] HU.

We proved and verified that an interior ROI can be exactly and stably reconstructed via the total variation (TV)/high-order TV (HOT) minimization if the ROI is piecewise constant or polynomial [22], [24], [27], [32], [39]–[41]. To improve the accuracy of iterative reconstruction, we recently derived a new re-projection scheme [42]. To accelerate the convergence, we employed the projected gradient method [43], and implemented a fast iterative scheme [44] for interior CT. To demonstrate the feasibility of interior CT, an ECG-gated CT scan was retrospectively selected on a GE Discovery CT750 HD scanner [41]. After pre-processing, we obtained a fan-beam sinogram in the ring-shaped omni-tomographic geometry. Over a full-scan range, 2200 projections were uniformly acquired, with 888 detector elements equi-angularly distributed for a field of view of 24.92 cm in radius. Each projection was truncated by discarding 300 data on each side of the detector array, focusing on an interior ROI of 8.70 cm in radius. It can be seen in Figure 6 that the interior reconstruction is in excellent agreement with the conventional FBP global reconstruction.

### Interior SPECT

**Figure 7:**
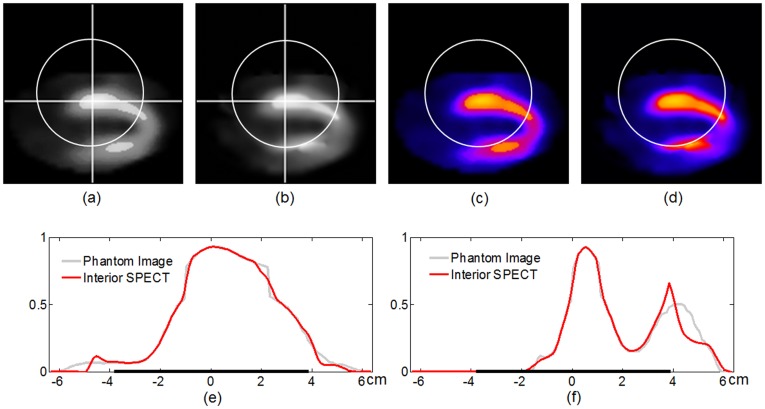
Interior SPECT reconstruction of a cardiac phantom. (a) An original SPECT ROI image of 128×128 pixels covering an area of 12.8×12.8 cm^2^, (b) an interior reconstruction using the HOT minimization algorithm with the attenuation background µ_0_ = 0.15 after 40 iterations, (c) and (d) the pseudo-color counterparts of (a) and (b) respectively. (e) and (f) The profiles along the horizontal and vertical white lines respectively in (a) and (b), where the thick lines on the horizontal axes indicate the ROI. The display window for (a) and (b) is [0, 1.0] in a normalized unit.

Inspired by interior tomography originally developed for CT, we proposed interior SPECT, from uniformly attenuated local projection data only through an ROI with a known sub-region in the ROI [45]. Then, we proved that interior SPECT reconstruction is theoretically unique from uniformly attenuated local projection data if an ROI is piecewise polynomial [28]. Actually, interior CT and interior SPECT have similar computational structures, with the major difference between the two modalities in the steepest gradient direction. In our numerical simulation, a SPECT cardiac perfusion image from the Internet was modified into a realistic 128×128 image phantom over an area of 128×128 mm^2^. A constant attenuating background 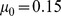 cm^−1^ was assumed on the standard patient support. An equi-spatial detector array was made of 78 detector elements, each of which was 1.0 mm in length. A full-scan was acquired consisting of 128 equi-angular projections. It can be seen in Figure 7 that interior SPECT produced excellent results, even if the piecewise polynomial model for n = 2 is not satisfied.

### Interior MRI

For omni-tomography, we use a local magnetic background field. This local magnetic field can be either homogeneous or inhomogeneous in principle, but the former is much easier to demonstrate feasibility and gain better performance. As long as the homogeneity of the background magnetic field holds over an ROI, classical MRI (including correction steps) methods [46] can be adapted for interior MRI [14].

As a prerequisite for working with a locally homogeneous main field, a scheme is needed to extract MR signals from an interior ROI. Here we propose a time-varying gradient method. Specifically, we can keep a gradient field zero at an ROI slice and vary the gradient field quickly outside that level. In this way, we can generate MR data only from the ROI, since magnetic iso-regions outside the ROI are incoherently excited and negligible. A similar strategy can be used to collect MR signals from a volume of interest. The key idea is to take advantage of regions with equal magnetic field strength, or “level sets”, in the background field for spatial localization. This approach accommodates a certain degree of field non-homogeneity, and is fundamentally different from the conventional MRI where we start with a globally homogeneous main magnetic field. Thus, our scheme reduces the size (and cost) of the main magnet.

In the following, we will focus on 2D interior MRI for simplicity. With the sole excitation of an ROI, we can use standard linear x and y gradient fields with slopes *G_x_* and *G_y_* respectively for spatial encoding. That is, the demodulated signal, assuming the uniformity of the receiver coil, is proportional to
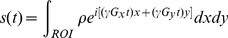
(1)where 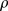 represents a 2D MR image to be reconstructed. On the discretized Cartesian grid, we rewrite the MR signal equation by
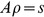
(2)where A is the system matrix discretized from Eq. (1). Under the least-square data fidelity and the TV regularization, interior MRI can be formulated as
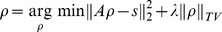
(3)where 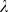 is the regularization parameter. Here we apply the split Bregman method [47] as an efficient solver of Eq. (3).

**Figure 8:**
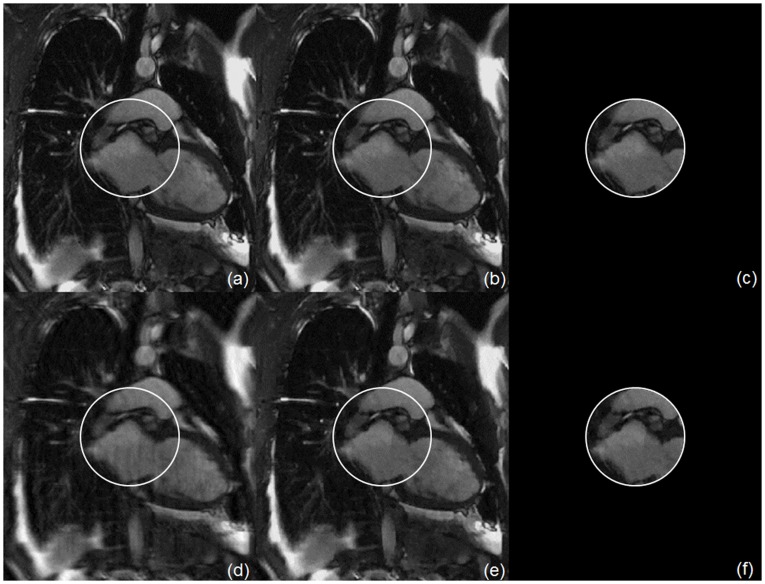
Interior MRI reconstruction of a cardiac image phantom. The top row (a)–(c) is from fully sampled data (100%), and the bottom row (d)–(f) is from randomly under-sampled data (25%) along the phase-encoding direction. The first column (a) and (d) is by the inverse fast Fourier transform (IFFT), the second column (b) and (e) by the TV minimization from global MR data, and the third column (c) and (f) by the TV minimization from interior MRI data.

We used an MRI cardiac image as the phantom. As shown in Figure 8, the proposed interior MRI method generated accurate interior reconstruction, based on a locally homogeneous main magnetic field. In this example, we compared the results with L2 regularization, TV regularization, and TV regularization for interior reconstruction with fully sampled k-space data and under-sampled k-space data respectively. In reference to the practical sampling pattern, we under-sampled the phase encoding direction and regularly sampled the frequency encoding direction.

### Unified Interior CT-MRI

Although various modalities have different contrast mechanisms, the physiological process to be reconstructed is the same. Consequently, there is strong correlation among these different types of data. To optimize the composite image quality, our unified omni-tomographic approach is based on inter-modality coherence, which is important prior information unavailable in the traditional reconstruction approach when image reconstruction is separately considered for each modality. In light of lowered sampling rate using temporal/spectral image coherence [48], [49], we hypothesize that inter-modality coherence can be utilized for omni-tomography to reduce data requirements further, while giving the same image quality.

In general, the multi-modal imaging model is
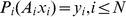
(4)where *P_i_*, *A_i_*, *x_i_*, and *y_i_* are an under-sampling operator, an imaging system matrix, an image, and data respectively, for the *i*th modality. Then, we have the matrix model
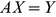
(5)where the ith column of X (Y) corresponds to xi (yi), and A should be understood in terms of Eq. (4).First, we have the rank-sparsity decomposition
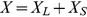
(6)where XL and XS are low-rank and sparse components respectively. Then, we have the optimization problem
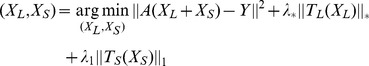
(7)where ||•||* is the nuclear norm to enforce the inter-modality coherence of XL after a transform TL with a parameter 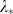, and ||•||1 is the L1 norm to promote the sparsity of XS after a transform TS with a parameter 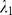. In this study, TL was the identity transform, and TS a linear framelet transform. Eq. (7) was solved using the split Bregman method [47].

**Figure 9:**
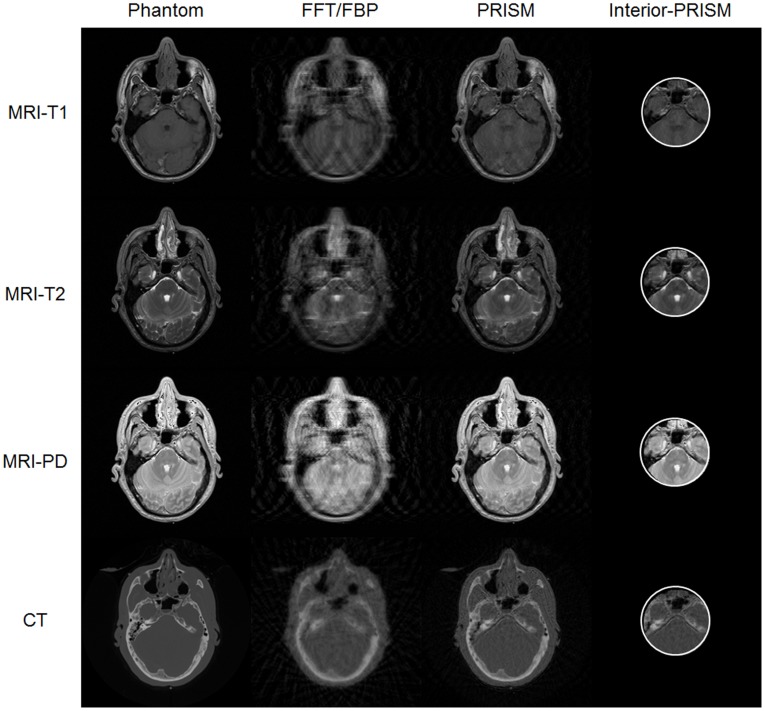
Unified CT-MRI reconstruction using inter-modality coherence. An MRI-CT head scan consisted of MR T1 (the 1^st^ column), T2 (the 2^nd^ column), proton density images (the 3^rd^ column), and a CT image (the 4^th^ column). The top row shows the phantom images, the middle row the images separately reconstructed using the conventional FFT or FBP method, and the bottom row the images simultaneously reconstructed in the unified rank-sparsity decomposition framework.

In this pilot study, we selected a set of MRI and CT head scans on the same human subject from the NIH Visible Human Project (http://www.nlm.nih.gov/research/visible), including MRI T1, T2, proton density, and CT images. The data were under-sampled with a factor of 8. The MR Cartesian k-space data were pseudo-randomly under-sampled along the phase encoding direction [50]. The fan-beam CT data were under-sampled using the dynamical strategy [49]. As shown in Figure 9, the unified reconstruction improved the image quality significantly. Clearly, this unified reconstruction framework can be extended to cover more imaging modalities in support of omni-tomography.

## Discussion

### Trend of Hybrid Imaging

Tomography is widely used for preclinical and clinical imaging to characterize morphology and to a limited extent physiology. Given current technology, we have been forced to accept several intrinsic limitations, especially the necessity to acquire images sequentially on the same subject. This time-wise separation impairs our ability to decipher correlated dynamic biological functions. For example, a cardiac infarct commonly begins with decreased perfusion, then tissue hypoxia and eventually cell death. These stages evolve in a continuum over a short period relative to the time to acquire multimodality data.

If technology could be developed to simultaneously image physiome of dynamic complexity with multiple modalities, rapidly evolving physiological processes will become transparent. Such processes have temporal evolution at many intervals, including sub-seconds, seconds, minutes or longer, and may or may not be reversible. A single session imaging is important to study processes such as ischemia, drug interactions, radiation effects, apoptosis, and many others. To some extent, this has partly been accomplished with PET-CT and MRI-PET systems. Although many of these multimodality imaging systems still acquire data sequentially, the delay in data acquisition is improved relative to single-modality predecessors.

With the Physiome Project, the need to simultaneously acquire and unify multimodal images has become more important than ever before. There are critical and immediate needs to remove the limitations inherent in today's tomographic imaging approaches to the extent that complex biological processes can be studied *in vivo* in real-time using multiple modalities. In a recent review article entitled “*Multimodality Imaging: Beyond PET/CT and SPECT/CT*” [18], Dr. Simon Cherry wrote that “*Multimodality imaging with PET/CT and SPECT/CT has become commonplace in clinical practice and in preclinical and basic medical research. Do other combinations of imaging modalities have a similar potential to impact medical science and clinical medicine? The combination of PET or SPECT with MRI is an area of active research at the present time, while other, perhaps less obvious combinations, including CT/MR and PET/optical also are being studied*.” Later, Dr. Cherry raised a question that could represent the thought processes of experts in the field: “*Is the fusion of PET and SPECT with CT the ultimate answer in multimodality imaging, or is it just the first example of a more general trend towards harnessing the complementary nature of the different modalities on integrated imaging platforms?*”.

Our answer is the tightest integration of all relevant modalities for fusion of information from each technology. We intend to reach this target by applying the latest insights from interior tomography to guide the design of the omni-tomographic system – A system that places the highest demands on the broadest array of imaging hardware and software. The logic seems clear that since subsets of imaging modalities are synergistic, the integration of all relevant modalities as a whole should add value above that of individual subsets. From this perspective, we can confidently expect that some form of omni-tomography is certain to be developed in the near future. This is consistent with past medical imaging innovations in that a major technical advancement can always find significant biomedical applications.

Major obstacles for grand fusion of imaging modalities are the gantry space limitation, the associated difficulties, and high cost. Omni-tomography can meet these challenges for three major reasons: (1) interior tomography can be accurately applied to most imaging modalities; (2) interior imaging is relevant in a majority of functional imaging studies; and (3) grand fusion will be cost effective when all or many imaging modalities must be used. An immediate advantage of interior imaging can be seen in how the problem of SPECT-MRI must be addressed. SPECT-MRI has two unique issues: interference to the magnetic field caused by a rotating camera head, and the induction of eddy currents in the camera head. Fortunately, interior SPECT will handle both issues simultaneously. With a smaller SPECT camera, the electromagnetic interference will be reduced, and the electromagnetic shielding design will be simplified. At the same time, the Eddy current will diminish in significance with better shielding for smaller detector heads. Additionally, an omni-tomographic scanner can contain a regular tomographic imager to provide a global reference.

Being consistent with the omni-tomography concept, we conceive three inter-related lines of grand fusion: (1) an architectural fusion of all or many imaging modalities into a single gantry; (2) a component fusion that packs all or many detectors into a single device or chip; and (3) a methodological fusion for data processing and image reconstruction in a unified framework, as exemplified in the above-described simulation of unified CT-MRI reconstruction.

### Push by Systems Biomedicine

At the beginning of this article, we mentioned the IUPS Physiome Project. Indeed, omni-tomography offers the best opportunity to observe well-registered tempo-spatial features in an unprecedented fashion. It may help reveal many unknown physiological, pathological, pharmaceutical and interventional interactions *in vivo*, significantly improving the sensitivity and specificity of basic research, diagnosis and intervention. This is the major hypothesis that can only be tested with a prototype omni-tomographic system.

Imaging technology development must target important biomedical problems. For example, biotechnology and bioinformatics have been developed to decode:

- Genomic/epigenetic signals at the DNA level associated with various forms of genomic signatures (*e.g*., single mutations, rare mutations, SNPs, copy number changes, indels, genomic instability index, *etc*.);
- Gene expressions at mRNA, miRNA, shRNA, gene, exon, and splicing levels, and their various functions;
- Protein expressions (protein complexes, metabolic features, *etc*.);
- Complicated interactions and networks among these players on multi-scales.

Many of these studies are currently limited to cells (tissue samples, cell lines, *etc*.) and rarely go beyond studies *in vitro*, yet imaging *in vivo* should facilitate or guide translation to organ, system, and body levels.

Systems biology should be a great driver for omni-tomography. Let us consider a biological subject as a system with inputs, circuitry with feedback loops, and outputs. Our omni-tomographic system, with the help of probes, can image some components of such a system in multiple dimensions (e.g., time, space, characteristics). One can imagine multiple ways in which this imaging system would be attractive and even indispensable. In the following, let us examine some potential applications of omni-tomography.

### Hope for Major Applications

Fluoroscopy guided cardiac catheterization has been the original clinical standard for identifying stenotic lesions in the coronary arteries. Recently, other diagnostic imaging techniques are being researched and applied for this application, such as CT, MRI, PET, SPECT, intra-coronary ultrasound, and optical coherence tomography. The latest emphasis is to improve understanding of pathobiology and genetics behind coronary artery diseases. There are demands for better diagnostic performance to achieve this goal. For example, the ability to model high risk atherosclerotic lesions would be clinically invaluable. The development of imaging methods and computational models is needed to identify and predict high-risk lesions that may rupture leading to coronary thrombosis and myocardial infarction. If our proposed system could be used to study preclinical infarction models, and predict outcomes, it would be instrumental for research and healthcare.

Oncological imaging already uses all the imaging modalities. Lung cancer imaging is a good example. CT defines air-tissue interfaces, detects nodules and tumors, and provides quantitatively accurate information. MRI measures airway functions with hyperpolarized Helium-3. PET and SPECT improve lung cancer diagnosis and staging with radiotracers. Omni-tomography could potentially quantify malignancy without biopsy. Also, a futuristic feature might use omni-tomographic data to create a knowledge depository linked to genetic/epigenetic information and patient histories.

The omni-tomographic system may be first developed for drug development in animal models, with aid of multimodality probes. This application is not subject to lengthy FDA approval, and will set a stage for translational research and clinical trials. Engineering probes visible to multiple modalities is a hot topic in the field of bio-nanotechnology, and will have a profound impact on drug development and molecular medicine [51]. Recently, exciting work was reported on a combination of multiple nano-millimeter-sized components to facilitate multimodality imaging and even enable new imaging modes [52].

It is acknowledged that not all imaging tasks can be reduced to interior imaging. To address this inherent ROI limitation, our interiorized omni-tomography approach may be extended for globalized omni-tomography. One idea is to simplify each data acquisition chain for sparse sampling that makes space for interlacing various imaging modalities. In a substantial sense, the above-described omni-tomographic imager is a special case of this more general plan. Further details are beyond the scope of this paper.

### Concluding Remark

Omni-tomography is a fresh concept with its interior reconstruction feasibility demonstrated for CT, SPECT, MRI, and other modalities. Although it may be more complex and costly than any conventional scanner, we expect a prototype can be built in the near future to demonstrate its instrumentation feasibility and biomedical utility. We share our excitement towards omni-tomography, and believe that it will find important applications especially for systems biomedicine. We are committed to working along this direction.

## Supporting Information

Video S1 Virtual reality video clip showing the ‘ring-shaped’ omni-tomographic system design.
(WMV)
